# The prodromes of Parkinson's disease

**DOI:** 10.1111/ejn.14269

**Published:** 2018-12-05

**Authors:** Richard Nathaniel Rees, Alastair John Noyce, Anette Schrag

**Affiliations:** ^1^ Department of Clinical and Movement Neurosciences Institute of Neurology University College London London UK; ^2^ Preventive Neurology Unit Wolfson Institute of Preventive Medicine Queen Mary University of London London UK

**Keywords:** anosmia, GBA, LRRK2, neurodegenerative disease, REM sleep behavioural disorder

## Abstract

Whilst the diagnosis of Parkinson's disease (PD) relies on the motor triad of bradykinesia, rigidity and tremor, the underlying pathological process starts many years before these signs are overt. In this prodromal phase of PD, a diverse range of non‐motor and motor features can occur. Individually they do not allow a diagnosis of PD, but when considered together, they reflect the gradual development of the clinical syndrome. Different subgroups within the prodromal phase may exist and reflect different underlying pathology. Here, we summarise the evidence on the prodromal phase of PD in patient groups at increased risk of PD with well described prodromal features: patients with idiopathic rapid eye movement sleep behaviour disorder, patients with idiopathic anosmia and families with monogenic mutations that are closely linked to PD pathology. In addition, we discuss the information on prodromal features from ongoing studies aimed at detecting prodromal PD in the general population. It is likely that better delineation of the clinical prodromes of PD and their progression in these high‐risk groups will improve understanding of the underlying pathophysiology.

**Abbreviations**
[^123^I]β‐CIT: [^123^I]β‐carboxymethoxy‐3β‐[4‐iodophenyl] tropane
BSIT: brief smell identification test
DAT‐SPECT: dopamine transporter single photon emission tomography
DLB: dementia with Lewy bodies
GBA: glucocerebrosidase
LRRK2: leucine‐rich repeat kinase 2
MDS: international Parkinson and movement disorder society
MOCA: montreal cognitive assessment
MRI: magnetic resonance imaging
MSA: multiple systems atrophy
PD: Parkinson's disease
PRIPS: prospective validation of risk factors for idiopathic Parkinson's syndromes
PSG: polysomnography
RBD: REM sleep behavioural disorder
REM: rapid eye movement
SNCA: α‐synuclein gene
SPECT: single photon emission tomography
TCS: transcranial sonography
TREND: Tübingen evaluation of risk factors for early detection of neurodegeneration
UMSARS: unified multiple systems atrophy rating scale
UPDRS: unified Parkinson's disease rating scale

## 1 INTRODUCTION

Parkinson's disease (PD) is diagnosed when bradykinesia occurs along with rigidity or tremor, with consideration of additional supporting and exclusionary features (Hughes, Daniel, Kilford, & Lees, 1992; Postuma, Berg, et al., 2015). However, patients frequently report having had symptoms for years before the diagnosis (Gaenslen et al., 2014) and database analyses have shown that a number of clinical features occur more frequently in patients with a later diagnosis of PD than controls (Schrag, Horsfall, Walters, Noyce, & Petersen, 2015)**.** Whilst it is now accepted that a long prodromal phase can exist, the progression of clinical features and the underlying pathological correlates during this phase are poorly understood, and reliably identifying people in this phase remains a challenge (Berg et al., 2015). Neuropathological studies suggest that at the time of diagnosis there has been significant neuronal loss in the substantia nigra (Braak et al., 2003; Greffard et al., 2006) and within 4 years of diagnosis, there is almost complete loss of dopamine terminals in the dorsal putamen (Kordower et al., 2013). Through extrapolation of these data, it has been estimated that the disease process, in the brain at least, has been developing for 5 to 10 years before diagnosis (Chu, Buchman, Olanow, & Kordower, 2018; Fearnley & Lees, 1991; Greffard et al., 2006). Understanding the clinical and pathophysiological aspects of this prodromal phase of PD is a key challenge in PD research to enable earlier diagnosis, investigation of the pathophysiological cascade and, ultimately, disease‐modifying treatment.

The Oxford English Dictionary defines a prodrome as “a symptom or sign, or a set of symptoms or signs, that is characteristic or premonitory of the onset of a disease; (also) an early phase of a disease in which its symptoms or signs are mild or non‐specific” (OED, 2018). Whilst this definition applies to what is currently known of the prodromal phase of PD, a range of different combinations of symptoms and clinical signs can occur. Individually they do not allow a diagnosis of PD, but when considered together, they reflect the gradual development of the clinical syndrome. Research criteria have been developed based on a combination of such features (Berg et al., 2015). However, the combination of different symptoms and signs may differ between individuals and may reflect different disease subgroups.

Here, we summarise the evidence on the prodromal phase of PD in patient groups at increased of PD with well described prodromal features: patients with idiopathic rapid eye movement (REM) sleep behaviour disorder (RBD), patients with idiopathic anosmia and families with monogenic mutations that are closely linked to PD pathology, in particular the Leucine‐rich repeat kinase 2 gene (*LRRK2*) and glucocerebrosidase gene (*GBA*). We also discuss the information on prodromal features from ongoing studies aimed at detecting prodromal PD in the general population.

## 2 REM SLEEP BEHAVIOUR DISORDER

RBD is a parasomnia in which there is loss of muscle atonia with accompanying enactment of vivid dreams during the REM phase of sleep. These dream enactments can include vocalisation (including shouting and swearing) and complex motor behaviours that can cause significant injury to the patient or their bed‐partner. It typically occurs in older men (mean age at onset 50–65). An accurate diagnosis does not rest on the history alone, but requires polysomnography (PSG), with EEG recording to exclude REM sleep‐related seizure disorders or other non‐REM parasomnias, and EMG to accurately record muscle tone during sleep (Boeve et al., 2007; Howell & Schenck, 2015).

RBD is closely linked to PD (and other synucleinopathies like dementia with Lewy bodies (DLB) and multiple system atrophy (MSA)) (Högl, Stefani, & Videnovic, 2017; Schenck, Boeve, & Mahowald, 2013): RBD frequently occurs in patients with PD, DLB or MSA with no prior sleep disorder (Boeve, Silber, Ferman, Lucas, & Parisi, 2001; Vetrugno et al., 2004). In addition, almost all people with idiopathic RBD develop one of these three conditions with extended follow‐up (Iranzo et al., 2014). Neuropathological studies of individuals with RBD and no diagnosis of other neurodegenerative disease reveal α‐synuclein deposits (Boeve et al., 2007; Vilas et al., 2016). Interestingly, in RBD the pathological changes are marked in the locus coeruleus and there is significant cholinergic and catecholaminergic loss as opposed to the predominant dopaminergic loss in PD (Dugger et al., 2012; Gaig & Tolosa, 2009), suggesting a possible alternative sequence of pathological events compared to PD without RBD.

PD and RBD also share similarities on imaging studies. MRI studies have shown very similar patterns of microstructural damage or altered diffusion patterns in RBD and PD (Barber, Klein, Mackay, & Hu, 2017; Heim, Krismer, De Marzi, & Seppi, 2017; Pyatigorskaya et al., 2017). Transcranial sonography (TCS) identifies increased echogenicity in the area of the substantia nigra in around 10% healthy controls, 90% of PD and 36%–50% RBD (Iranzo et al., 2010; Li, He, Liu, & Chen, 2016). Dopamine transporter single photon emission tomography (DAT‐SPECT) in RBD cohorts point towards this test's utility as a marker of impending conversion to overt Parkinsonism (Högl et al., 2017).

As well as the sleep disorder, the RBD prodrome of PD includes objective motor and non‐motor deficits, with impaired balance (Chen et al., 2014), abnormal gait (Alibiglou, Videnovic, Planetta, Vaillancourt, & MacKinnon, 2016; Videnovic et al., 2013) and other fine and gross motor slowing (Postuma, Gagnon, Vendette, & Montplaisir, 2009). Postuma et al. demonstrated a significant motor difference in individuals with RBD who went on to develop PD compared to those who remained with “isolated RBD”: through longitudinal semi‐quantitative motor assessments, the two groups deviated around 4 years before a PD diagnosis in UPDRS motor scores, rigidity, gait difficulties and limb bradykinesia. With more objective markers, such as the Purdue pegboard, motor differences were detectable over 8 years prior to diagnosis (Postuma, Lang, Gagnon, Pelletier, & Montplaisir, 2012). In another 10‐year prospective study in an RBD cohort, abnormal colour vision, anosmia and mild motor dysfunction were all strongly predictive of conversion to PD (Postuma, Gagnon, Bertrand, Génier Marchand, & Montplaisir, 2015). Erectile difficulty and constipation were also more common in the baseline assessments of the same cohort (Postuma et al., 2009). Fereshtehnejad et al. also found that there was a highly significant difference in olfactory loss in the RBD cohort who converted to PD (75%) compared to those who did not (48%, *p* < 0.004) (Fereshtehnejad et al., 2017). Further linking RBD and anosmia as prodromal markers, Mahlknecht et al. found that patients with RBD who converted to PD or DLB within 4 years had significantly worse olfactory function at baseline than healthy controls, and were in the same range of smell performance as the PD patients (Mahlknecht et al., 2015). However, although anosmia is more common in RBD cases than controls, it does not appear to be a progressive feature in RBD, and in a study by Iranzo et al. (2013), the differences in smell between the RBD and control groups did not change over 4 years of longitudinal follow‐up. Cognitive dysfunction has been recognised in RBD, although whether this represents the prodromal phase of PD or DLB is a matter of debate (Génier Marchand, Montplaisir, Postuma, Rahayel, & Gagnon, 2017; Youn et al., 2016), and patients with concurrent PD and RBD have significantly more cognitive dysfunction than those with PD without RBD (Lin & Chen, 2018). When applying the MDS prodromal criteria, which combine a range of clinical and biomarker findings, to a PSG‐confirmed RBD cohort of whom nearly 40% converted to established PD, the sensitivity of the criteria in the year prior to the diagnosis of PD was 100%; excluding RBD from the analysis (with the associated likelihood ratio of 130), the specificity was 96.4% with a positive predictive ratio of 87.5%. The sensitivity, however, fell to 14.6% without RBD (Fereshtehnejad et al., 2017). Together these findings suggest that patients with RBD also develop a variety of other prodromal features before converting to clinical PD. However, with a highly variable duration of RBD prior to conversion to PD, the determinants and markers of progression and conversion from RBD to PD remain to be finalised.

## 3 IDIOPATHIC ANOSMIA

Chronic anosmia or hyposmia can be caused by a variety of conditions: congenital, post‐traumatic, post‐infectious, idiopathic and related to neurodegeneration (Boesveldt et al., 2017). Anosmia is a common finding, affecting up to 20% of the population and is associated with ageing (Boesveldt et al., 2017). Anosmia is also a common finding in PD, with the majority of PD patients having impaired or absent smell (Ross et al., 2008). Underpinning these observations is neuropathological evidence that suggests that the olfactory bulb is affected early in the disease process, creating an anatomical link between the two conditions (Braak et al., 2003; Pearce, Hawkes, & Daniel, 1995; Ubeda‐Bañon, Saiz‐Sanchez, de la Rosa‐Prieto, & Martinez‐Marcos, 2014).

Patients commonly report that they lost their sense of smell years before the diagnosis of PD (Haehner, Hummel, & Reichmann, 2009), and there is considerable evidence for olfactory loss as a prodromal feature of PD. In a study of 361 individuals with a first degree relative with PD and no diagnosed neuropsychological or olfactory disorder, those with impaired olfaction (defined as the lowest 10% Z‐scores by age group and gender) had an increased risk of developing PD after 2 years of follow up. The hyposmic group also had an increased rate of abnormal [^123^I]β‐CIT SPECT compared to those with normal smell (Ponsen et al., 2004). In the Health, Aging and Body Composition study, poor olfaction using the brief smell identification test (BSIT) predicted the onset of PD with a hazard ratio of 4.8 for the lowest tertile (≤8/12 correct responses) compared to the highest tertile (≥11/12 correct responses) adjusting for sex and race over an average follow up of 9.8 years (Chen et al., 2017). In the Honolulu‐Asia Aging Study, smell loss (again tested with the BSIT — with a lower cut‐off of ≤5/12 correct responses) was associated with a 5‐fold increased risk of PD at 4 years follow‐up, when adjusted for age and other factors that affect PD risk (cigarette smoking, coffee consumption and constipation) (Ross et al., 2008). Interestingly, they found that this increased risk was not seen at follow‐up more than 4 years without conversion to PD. This raises the possibility that anosmia as part of a Parkinson's prodrome may only develop within 4 years of manifestation of PD.

The Parkinson At‐Risk Study (PARS) has utilised this increased risk of PD in people with olfactory loss to further study other aspects of the prodrome of PD. This multicentre North American study (Siderowf et al., 2012) recruited 669 adults >50 years with hyposmia (defined as <15^th^ centile using the UPSIT) and 4330 with intact smell. At baseline, constipation (defined as <1 bowel movement per day) was more frequent in those with hyposmia but common in both groups (21% vs. 16%) as were anxiety (state 19% vs. 14%, trait 23% vs. 17%) and depression (19% vs. 11%). Reported features of RBD were also more common in those with hyposmia (12% vs. 7% for ≥1 limb/body movement per week; 3% vs. 1% violent movements/week). In follow‐up reports, whilst the hyposmic cohort did not have worse UPDRS scores than the normosmic controls, a significantly greater proportion had DAT deficit using [^123^I]β‐CIT SPECT (11% vs. 1%). Of the hyposmics with abnormal DAT scans, other prodromal symptoms were common: RBD, subtle motor symptoms or constipation (Jennings et al., 2014). Furthermore, in the hyposmic group, DAT deficit was strongly associated with conversion to PD within 4 years with a relative risk of 17.5 compared to those with indeterminate or no DAT deficit (Jennings et al., 2017).

In another study using olfaction as one key identifier, the Tübingen Evaluation of Risk Factors for Early Detection of Neurodegeneration (TREND) study has followed nearly 700 “healthy” older adults (50–85 years) with prodromal markers (impaired olfaction, depression or RBD) with biannual assessments. Those with anosmia had significantly fewer other prodromal features than the other two groups at baseline. Marked differences were reported in the rates of constipation (7% in the anosmic group compared to 22% and 25% in the RBD and depression groups), insomnia other than RBD (34% vs. 65% and 53%), visuospatial perception (2.2% vs. 12% and 14%), orthostatic hypotension (13% vs. 33% and 25%) and cognitive complaints (bradyphrenia: 24% vs. 36% and 46%, word recall: 37% vs. 60% and 53%) (Gaenslen et al., 2014). Of the 10 participants in the TREND study who converted to PD within 6 years, nine had hyposmia at baseline, and all had probable (not PSG‐confirmed) RBD (although it should be noted that this study was enriched for both these markers).

Whilst olfactory loss is clearly an important component of the prodrome of PD in a large proportion of patients with PD, there is a (potentially large) proportion of individuals with idiopathic anosmia not linked to neurodegeneration. The above studies provide important information on anosmia in the prodrome of PD but further examination and longitudinal assessment of people with idiopathic anosmia evaluated for all known causes of hyposmia will be needed to determine the boundaries and characteristics of anosmia as a prodromal PD phenotype.

Overall, however, whilst not all patients with PD have hyposmia or RBD even when the diagnosis is established (Haehner et al., 2009; Zhang, Sun, Wang, Tang, & Xie, 2017), both the detection of RBD and hyposmia offers the opportunity to create clinical cohorts to study the biological, pathological and biomarker correlates between RBD or idiopathic anosmia and neurodegeneration.

## 4 LRRK2

Although only a small proportion of patients with PD have a monogenic cause of PD, compared to other risk factors family history has one of the strongest associations with PD (Noyce et al., 2012). A number of cohort studies of PD therefore also include an arm to longitudinally study first‐degree family members of patients with PD, including the PPMI, OPDC and Tracking Parkinson's/PRoBaND studies (Malek et al., 2015; Parkinson Progression Marker Initiative, 2011; Szewczyk‐Krolikowski et al., 2014). However, whilst only a small minority of individuals with PD have a monogenic cause of PD, those with mutations known to confer an increased risk of PD provide the clearest information on the development of prodromal features. In the US and European studies, the commonest cause of autosomal dominant PD is a mutation in the *LRRK2* gene, accounting for about 1% of all PD cases (Healy et al., 2008). This is a complex gene whose role in neurodegeneration is not completely understood (Kalia et al., 2015). Penetrance is incomplete and carriers of the mutation have an age‐dependent risk of PD that reaches around 25%–42% at 80 years (Lee et al., 2017; Marder et al., 2015). Once manifest, the motor features of *LRRK2*‐PD are largely indistinguishable from idiopathic PD, and examination of carriers of *LRRK2* mutations therefore provide a useful model for prodromal PD. In the last few years, several longitudinal cohort studies of *LRRK2* carriers have been published (Mestre et al., 2018; Mirelman et al., 2015, 2018; Pont‐Sunyer et al., 2017; Sierra et al., 2013, 2017). However, unlike in “idiopathic” PD, anosmia has been reported to be infrequent in *LRRK2* PD cases (Marras et al., 2016) and also in *LRRK2* carriers (Mestre et al., 2018; Mirelman et al., 2015; Pont‐Sunyer et al., 2017). On the other hand, *LRRK2* carriers had more errors on the Farnsworth‐Munsell 100 Hue test of colour discrimination than healthy controls, and *LRRK2* PD patients compared to idiopathic PD patients (Marras et al., 2016). One study reported that constipation was a more common feature among *LRRK2* carriers than controls (Mirelman et al., 2018), but other autonomic, affective and cognitive functions were not significantly different across most studies reviewed. Motor dysfunction measured on the UPDRS III was not significantly different in a Spanish *LRRK2* cohort in those who converted to PD compared to non‐converters at baseline, although in those that went on to convert to PD within 4 years there was significant worsening of the UPDRS III scores from a mean of 9.3 at baseline to 25 compared to 0.8 to 1.6 in the non‐converting group (although the number of converters was small) (Sierra et al., 2017).

With the generation of a mouse model with *LRRK2* R1441C mutation, Giesert et al. have shown prodromal features in old‐age mice in several measures of gait analysis, anxiety tests and olfaction (Giesert et al., 2017). This work shows that animal models of the prodromal phase of PD can be established, and as the biology of other prodromes is revealed, laboratory models can be tailored to explore these phenotypes.

## 5 GBA

Glucocerebrosidase is a lysosomal enzyme encoded by the *GBA* gene. Homozygous *GBA* mutations cause type 1 Gaucher's disease (GD), the commonest lysosomal storage disorder (McNeill et al., 2012), and patients with GD are at increased risk of PD. About 5%–10% of PD patients have mutations in the *GBA1* gene, with a higher proportion in certain populations (particularly Ashkenazi Jews) (Schapira, 2015). The risk of conversion to PD in heterozygous carriers of a genetic mutation is similar to *LRRK2* carriers, with approximately 30% developing PD at 80 years (Anheim et al., 2012). This risk lies between that associated with rare but highly pathogenic mutations (such as in *LRRK2, SNCA* etc.) and mutations that are common but confer minimal additional risk influence (as detected through genome‐wide association studies). In patients with established PD who are heterozygous for *GBA* mutations the clinical features are similar to those with idiopathic PD, but a slightly earlier onset has been reported, and non‐motor symptoms including RBD and cognitive impairment occur more commonly and earlier than in idiopathic PD (Balestrino & Schapira, 2018). Compared to healthy controls, both GD patients and non‐GD carriers of GBA have slightly worse smell, cognition and motor impairment on the UPDRS III motor scale (Gatto et al., 2016; McNeill et al., 2012). In GBA carriers without established PD, Beavan et al. reported not only a higher rate of familial occurrence of PD than controls (GD 16.7%, GBA heterozygous 7.1%, controls 0% (*p* = 0.3)), but also worse olfactory function, more autonomic features (using the Unified Multiple Systems Atrophy Rating scale (UMSARS)), worse cognitive scores (using the Montreal Cognitive Assessment) and worse UPDRS II and III scores with significant evidence of progression over 2 years (Beavan et al., 2015). Ongoing follow‐up of this cohort will be essential to examine progression rates from the onset of the prodromal phase through to established PD.

## 6 GENERAL POPULATION

Several studies are also aiming to identify people with prodromal PD in the general population in order to more robustly define the prodromal features of PD outside of selected risk groups. Using different methods, these studies aim to enrich the general population for risk of PD using combinations of a variety of non‐motor and motor features, clinical features, and imaging biomarkers (DAT‐SPECT, TCS and/or MRI). These studies include the Prospective validation of Risk factors for Idiopathic Parkinson's Syndromes (PRIPS) study (Berg et al., 2013), TREND (Gaenslen et al., 2014) and PREDICT‐PD (Noyce et al., 2014). Whilst only longitudinal follow‐up will determine the association of the tested variables with development of clinical PD, preliminary results are already available. In a combined analysis of the TREND and PRIPS studies, those converting to PD and those with high probability according to the MDS research criteria for prodromal PD had higher rates than those who did not of RBD, anosmia, mild motor symptoms, constipation and erectile dysfunction (Pilotto et al., 2017). The PREDICT‐PD study uses an online approach to identify increased risk of PD using a combination of risk and prodromal factors. RBD, anosmia and motor dysfunction as well as abnormalities on DAT‐SPECT and TCS were significantly greater in the higher than the lower‐risk group (Noyce et al., 2017). These general population cohorts are likely to represent an amalgamation of different patient groups and will provide insight into the heterogeneity of PD even in this prodromal phase.

## 7 CONCLUSIONS

Retrospective studies and examination of newly diagnosed patients with PD have suggested a long prodromal phase of PD, beginning several years before definite features of clinical PD. There is now increasing information from cross‐sectional and prospective studies in cohorts at increased risk of PD, particularly those with RBD and anosmia and carriers of PD‐related mutations. In addition, approaches to combine several risk factors are increasingly providing insight into the identification and presentation of those in the earliest phases of PD. It is likely that better recognition of the clinical prodromes of PD will provide further understanding of their underlying pathophysiology.

## CONFLICT OF INTEREST

The authors state that they have no conflicts of interest.

## AUTHOR CONTRIBUTIONS

RNR devised the scope and outline of the review, wrote the first draft and studied the literature. AJN and AS critically reviewed and revised the manuscript, and provided additional interpretation and analysis. All authors give final approval for the paper to be published and are accountable for all aspects of the work.
