# Supplementary material for: The prodromes of Parkinson's disease
Source: Eur J Neurosci. 2018 Dec 5;49(3):320–7. doi: 10.1111/ejn.14269 (PMC6492156; doi:10.1111/ejn.14269)
Supplement: Supplementary file 1 [file EJN-49-320-s001.docx]

The Prodromes of Parkinson’s disease

Schrag, Anette ; Rees, Richard; Noyce, Alastair

_____________________________________________________________________________________

| Review timeline: | Submission date: 25/05/18  Editorial Decision: Major Revision  Revision Received: 26/09/18  Accepted: 08/11/18 |  |
| --- | --- | --- |
|  |  |  |

Editor: Paul Bolam

Reviewer 1: Ron Postuma

Reviewer 2: Wolfgang Oertel

_____________________________________________________________________________________

1st Editorial Decision	02/07/18

_____________________________________________________________________________________

The Prodromes of Parkinson’s disease 

Dear Dr. Schrag, 

Your manuscript has been reviewed by two external reviewers as well as by the Editorial team.   

As you can see, both reviewers consider that your review is interesting and important and we consider it a fitting contribution to the Special Issue in memory of Tom. Both however, raise a series of issues that will need to be addressed in a revised version of your manuscript.  Reviewer 1 is particularly concerned about your conclusions relating to the anosmia prodrome.  This will probably need adjusting and will need the removal of Table 1 (as also suggested by Reviewer 2).  If you could include a section on the basic science as suggested by Reviewer 2, it would be beneficial to the general readership of EJN.  Please carefully address each of the points that they have raised.   

We also note the following points that need to be addressed in the revised version. 
- Please provide a running title.   
- Please convert in-text references and reference list to EJN style. 
- Please provide a list of abbreviations, a COI statement and author contributions 

When revising the manuscript, please embolden or underline major changes to the text so they are easily identifiable and DO NOT leave 'track change' formatting marks in your paper.  Please ensure that you provide a text and a figure file for the Graphical Abstract (as detailed in the instructions below).  When carrying out your revisions please refer to the checklist below and visit the EJN author guidelines at www.ejneuroscience.org 

When finalized, please upload your complete revised manuscript onto the website, as a Word file (.doc, or .docx).  Please also ensure that a complete set of tables and figures is included as separate files, even if these have not changed from the originals.  At this stage it is necessary to provide high resolution figures.  Please see important instructions below. 

Please go into https://mc.manuscriptcentral.com/ejn - Author Centre - manuscripts with decisions where you will find a 'create a revision' link under 'actions'. We ask that you please indicate the way in which you have responded to the points raised by the Editors and Reviewers in a letter. Please upload this response letter as a separate Word (.doc or PDF) file using the file designation "Authors' Response to Reviewers" when uploading your manuscript files.  Please DO NOT submit your revised manuscript as a new one.  Also, please note that only the  Author who submitted the original version of the manuscript should submit a revised version. 

If you are able to respond fully to the points raised, we would be pleased to receive a revision of your paper within 12 weeks. 

Thank you for your support of this Special Issue of EJN. 

Best wishes, 

Paul Bolam & John Foxe 
co-Editors in Chief, EJN 


Reviews: 

Reviewer: 1 

Comments to the Author 
Whereas I personally agree that there are likely to be different subtypes of prodromal PD, there is a major logical error that runs through the paper, namely a confounding of subtype with specificity.  This is most evident in the olfactory section, so I'll illustrate it there: 

When comparing olfaction to the other prodromal symptoms, it is noted that patients in the TREND study who were identified with anosmia had a lower prevalence of many non-motor and motor signs.  Therefore the conclusion is that the anosmia prodrome is milder.  This misses the most obvious alternate explanation - the anosmic patients have fewer features because anosmia is non-specific, and so most of the patients don't actually have prodromal PD.  If >80% of RBD patients will develop PD, but only 10-20% of anosmics will, then obviously the prevalence of other prodromal symptoms will be radically different. 
- the only way to establish that the prodrome is truly different is to look specifically in the two groups among patients who actually developed PD (e.g. anosmics to PD vs. RBD to PD), and then compare their prodromes directly.  So far, such a backwards look has only been done for RBD, so the comparison cannot be made, and no conclusions can be drawn. 
- this means that all of the content of Table 1 is a guess, at best, and probably should be removed completely.  Most of the evidence for a different prodrome rests on this, and so the entire conclusion of the paper has not been supported. 

On the other hand, one could certainly draw inferences that the subtype of LRRK2 and GBA are very different, as they have predictive values that mostly match.  Probably, the best evidence, however, is that they are such different diseases once PD starts, particularly with regards to cognitive impairment and other non-motor manifestations. 

Not sure how to correct this.  If this is an invited review, I could simply suggest that the paper raise the point that there may be different prodromes and encourage readers to think about this issue,  but concentrate on describing the four prodromal states that we have so far (of course mentioning the effect of specificity). 

Other specific points 

The RBD section was accurate, but then the conclusion was that the RBD prodromal subtype is relatively aggressive / rapidly progressing.  This does not follow from the discussion before, and I don't think this is an accurate statement, as the mean onset between RBD and PD is 13 years.  Its hard to think of such a long interval as indicating an aggressive course. This statement may be a consequence of the mixup noted earlier (all of the RBD have prodromal PD, so they seem worse than the other cohorts).   It is true that the PD that develops out of RBD is of generally poor prognosis, but that is not the same thing. 

With regards to cognitive dysfunction as a prodrome of either PD or DLB, it should be noted that the diagnoses may not be mutually exclusive.  This means that the concern raised becomes less relevant.   

Regarding anosmia, studies estimate that 70-80% of PD patients have anosmia at baseline (very different than RBD with 25% and GBA/LRRK-2 with 1-3%).  Moreover, essentially all of the RBD and GBA patients have olfactory loss as part of their prodrome at some point.  If almost all PD patients have an anosmic prodrome, then is this really a subtype? 

For LRRK2, the phenoconversion rate is listed as 80% up to age 80.  Most of the recent studies are finding much lower penetrance than that.  For example, Marder Neurology 2015 finds 26% penetrance at age 80 in AJ, and Lee 2017 Movement disorders finds a penetrance of 42.5% in non-Jews (the authors of this study list this as among the highest penetrance estimates).  In fact, the penetrance may be very close to GBA once DLB is included (which allows a nice comparison of GBA and LRRK2). 

It is stated that the clinical features of LRRK2-PD are indistinguishable from clinical PD.   It should probably be corrected to motor features; as is pointed out later, the non-motor features can be strikingly different. 

It was stated that 5-10% of PD patients have GBA mutations; doesn't that make the previous statement that LRRK-2 is the commonest gene mutated incorrect (since they actually have similar penetrance)? 

Minor spelling - Purdue pegboard, not Perdue 


Reviewer: 2 

Comments to the Author 
The  manuscript reviews  at the descriptive level prodromal PD - by summarizing clinical symptoms and  signs which may  be suitable for classifying prodromal PD into subgroups. 
The authors subdivide prodromal PD in 4 subgroups (see below). They present characteristics of these 4 mentioned prodromal “ at risk populations “ and their interdependence with  each other and/ or with other prodromal characteristic parkinsonian features like subtle motor symptoms, pathological DAT-scan-results, autonomic deficits and cognitive and psychological impairment or symptoms. The quoted studies in this context addressing the early prodromal detection of PD are results of different prospective epidemiological designs, like: the Health, Aging and Body Composition Study, the Honolulu-Asia Aging study, the PARS ( Parkinson At Risk Study: multicenter North American study); the TREND study (Tübingen Evaluation of Risk Factors for Early Detection of Neurodegeneration); the PRIPS (Prospective validation of Risk factors for Idiopathic Parkinson’s Syndrome) and the PREDICT PD-study (identifying risk of Parkinson's disease in the community). 

The different manifestations of motor and non-motor symptoms of the 4 most important “at-risk” -cohorts for prodromal stages of PD (RBD; Anosmia; GBA- and LRRK2-mutations) are summarized in a table with  a score from 1-3. This table oversimplifies the clinical situation: RBD per definition has no motor impairment according to the UK brain bank criteria. The research published in this respect picks up subtle motor impairment – clinically hardly visible. 
A substantial proportion of RBD has normosmia. 

It is recommended to delete the table. It is misleading. 

The authors conclude: RBD represents the most aggressive and progressive form in respect to conversion into PD -  by looking at motor and non-motor features. In contrary, LRRK2 appears to present a benign prodromal progression towards conversion into PD.                                                                                                                                                                                                                                                                                                                                                                                                                                                                                                                                                                           
This article is submitted as contribution to a special issue addressing neuroscientists. 
The authors fail to write an article which could attract the target readership. This may have not been the task of the authors as outlined by the editor: 
There are no referals to basic science aspects, there is no attempt to compare animal models with the clinical situation. There are no proposals, how a neuroscientist should and could get involved in neuroscientific projects which may assist solving open clinical questions of prodromal PD. 
Further points: 
In the introduction the authors propose – this sounds as if the authors present a new theory – they summarize the present level of evidence. Please do not use “propose”. 
The article contains imprecise statements: 
The statement of reference 7 is not correct:  4 years after the diagnosis there are still nigrostriatal terminals detectable in the striatum. The authors should consult longterm follow up studies on early PD such as the PPMI data set. 
The statement that there Is a predominant loss of dopaminergic neurons in PD in comparison to RBD is of course correct . There is – however - a marked loss of neurons in the LC in PD post mortem (not specified which subgroup was investigated post mortem) – so it is questionable if a PD form exists, in which the LC is hardly affected and at the same time the substantia nigra is markedly degenerated. 
Prodromal cohorts are subdivided into 4 “specific at-risk-populations”: 
- REM-sleep-behavior disorders (RBD) 
- Anosmia/Hyposmia 
- Gene carrier for LRRK2-mutation and 
- Gene carrier for GBA-mutation. 
The first (RBD) and two genetic forms (LRRK2, GBA) are accepted prodromal stages pf PD. 
The strength of the article is the summary of the clinical features of the two genetic forms – as experimental research projects on the role of the two genes in the etiopathogenesis of PD can be designed. 
In contrast the two other subgroups are defined by a clinical phenotype  - as correctly stated by the authors. Therefore it is difficult to mirror these two prodromal subtypes into the neuroscientific area – as the cause(s) behind these phenotypes may be multiple (correctly stated by the authors). Here I miss a short discussion on the challenges neuroscientists face when they aim to establish prodromal models of PD in the laboratory. 
The definition of a subgroup “anosmia/hyposmia”  of prodromal PD is not convincing. They may define this group differently  but taken hyposmia as  a lead parameter for defining a prodromal group mirrors the shortcomings of the research performed in prodromal PD – nothing else. 
They do not discuss the issue of subclinical RBD and the fact that most likely the present screening procedures fail to discover female RBD patients. 
The authors correctly state, that RBD can only be diagnosed by polysomnography. They quote several large cohort trials (TREND, PARS and others) in which  - at least to my knowledge - the diagnosis of RBD was made by using screening questionnaires. So the article gives the impression that these publications give valid data on the comorbidity of hyposmia and properly diagnosed RBD in prodromal PD. To avoid this misunderstanding the authors should comment on the quality of the data (methodology) in the respective  articles. 
The authors should incorporate the data of Mahlknecht et al. 
Minor comments 
Page 4, line 12: The word  risk is missing 
Page 10:  converter with n 
The word  IN is missing before the word LRRK2

Reviewer 2:

Review of the Article: The Prodromes of Parkinson’s disease Journal: European Journal of Neuroscience

Recommendation. MAJOR REVISION

Or the authors clearly state the limitations of the article, that it is a purely descriptive manuscript – which still should be substantially improved. Comment to the editor: This manuscript is part of a special issue (I assume on alpha-synucleinopathies or Parkinson disease). The readership are basic neuroscientists . It may be that the editor(s) of the special issue has/ve requested a manuscript which entirely focusses on the clinical descriptive level of prodromal PD. The reviewer does not know the titles of the other articles – for example: is there an article which addresses the different theories of prodromal PD (prion-like spreading theory, Braak hypothesis and others). The table - although in principle a useful attempt - should be deleted. It oversimplifies the situation. It may be that there is another article, which bridges the clinical aspects of prodromal PD or prodromal alpha-synucleinopathies with experimental laboratory research (for example animal models). The article does not outline the theories behind prodromal PD, it does not tell the neuroscientists what the clinician would like to have investigated by neuroscientists I accept, that the scope and length of the article may limit the depth of discussion of the issues mentioned and critized below. The article is written by clinicians (neurologists), not clinician scientists. The authors are definitely not experts in prodromal PD. Commment to the authors: The manuscript reviews at the descriptive level prodromal PD - by summarizing clinical symptoms and signs which may be suitable for classifying prodromal PD into subgroups. The authors subdivide prodromal PD in 4 subgroups (see below). They present characteristics of these 4 mentioned prodromal “ at risk populations “ and their interdependence with each other and/ or with other prodromal characteristic parkinsonian features like subtle motor symptoms, pathological DAT-scan-results, autonomic deficits and cognitive and psychological impairment or symptoms. The quoted studies in this context addressing the early prodromal detection of PD are results of different prospective epidemiological designs, like: the Health, Aging and Body Composition Study, the Honolulu-Asia Aging study, the PARS ( Parkinson At Risk Study: multicenter North American study); the TREND study (Tübingen Evaluation of Risk Factors for Early Detection of Neurodegeneration); the PRIPS (Prospective validation of Risk factors for Idiopathic Parkinson’s Syndrome) and the PREDICT PD-study (identifying risk of Parkinson's disease in the community). The different manifestations of motor and non-motor symptoms of the 4 most important “at-risk” - cohorts for prodromal stages of PD (RBD; Anosmia; GBA- and LRRK2-mutations) are summarized in a table with a score from 1-3. This table oversimplifies the clinical situation: RBD per definition has no motor impairment according to the UK brain bank criteria. The research published in this respect picks up subtle motor impairment – clinically hardly visible. A substantial proportion of RBD has normosmia. It is recommended to delete the table. It is misleading. The authors conclude: RBD represents the most aggressive and progressive form in respect to conversion into PD - by looking at motor and non-motor features. In contrary, LRRK2 appears to present a benign prodromal progression towards conversion into PD. This article is submitted as contribution to a special issue addressing neuroscientists. The authors fail to write an article which could attract the target readership. This may have not been the task of the authors as outlined by the editor: There are no referals to basic science aspects, there is no attempt to compare animal models with the clinical situation. There are no proposals, how a neuroscientist should and could get involved in neuroscientific projects which may assist solving open clinical questions of prodromal PD. Further points: In the introduction the authors propose – this sounds as if the authors present a new theory – they summarize the present level of evidence. Please do not use “propose”. The article contains imprecise statements: The statement of reference 7 is not correct: 4 years after the diagnosis there are still nigrostriatal terminals detectable in the striatum. The authors should consult longterm follow up studies on early PD such as the PPMI data set. The statement that there Is a predominant loss of dopaminergic neurons in PD in comparison to RBD is of course correct . There is – however - a marked loss of neurons in the LC in PD post mortem (not specified which subgroup was investigated post mortem) – so it is questionable if a PD form exists, in which the LC is hardly affected and at the same time the substantia nigra is markedly degenerated. Prodromal cohorts are subdivided into 4 “specific at-risk-populations”: - REM-sleep-behavior disorders (RBD) - Anosmia/Hyposmia - Gene carrier for LRRK2-mutation and - Gene carrier for GBA-mutation. The first (RBD) and two genetic forms (LRRK2, GBA) are accepted prodromal stages pf PD. The strength of the article is the summary of the clinical features of the two genetic forms – as experimental research projects on the role of the two genes in the etiopathogenesis of PD can be designed. In contrast the two other subgroups are defined by a clinical phenotype - as correctly stated by the authors. Therefore it is difficult to mirror these two prodromal subtypes into the neuroscientific area – as the cause(s) behind these phenotypes may be multiple (correctly stated by the authors). Here I miss a short discussion on the challenges neuroscientists face when they aim to establish prodromal models of PD in the laboratory. The definition of a subgroup “anosmia/hyposmia” of prodromal PD is not convincing. They may define this group differently but taken hyposmia as a lead parameter for defining a prodromal group mirrors the shortcomings of the research performed in prodromal PD – nothing else. They do not discuss the issue of subclinical RBD and the fact that most likely the present screening procedures fail to discover female RBD patients. The authors correctly state, that RBD can only be diagnosed by polysomnography. They quote several large cohort trials (TREND, PARS and others) in which - at least to my knowledge - the diagnosis of RBD was made by using screening questionnaires. So the article gives the impression that these publications give valid data on the comorbidity of hyposmia and properly diagnosed RBD in prodromal PD. To avoid this misunderstanding the authors should comment on the quality of the data (methodology) in the respective articles. The authors should incorporate the data of Mahlknecht et al. Minor comments Page 4, line 12: The word risk is missing Page 10: converter with n The word IN is missing before the word LRRK2

____________________________________________________________________________________

Authors’ Response	26/09/18

_____________________________________________________________________________________

Response to reviewers

We agree with the reviewers comments and have significantly revised the manuscript accordingly. Specifically, we have adjusted our conclusions relating to the anosmia prodrome and removed Table 1. We have also include a section on the basic science for basic neuroscience researchers as suggested by Reviewer 2,

Reviewer 1

We agree with the comments regarding the specificity of anosmia for PD and have altered the manuscript accordingly (see page 10).

We have removed the table.

We have included a caveat regarding the RBD prodrome (p7)

We have amended the penetrance data to reflect the work by the MJF LRRK2 consortium.

We have altered the wording regarding the LRRK2 established PD phenotype.

In light of the amendments to the prevalence of LRRK2, we have amended the prevalence comparison between LRRK2 and GBA. (p12)

Purdue spelling changed.

Reviewer 2

We have removed the table.

We have modified our aims – to present a descriptive outline of different cohorts with the prodromal features, but without any direct comparison, given the paucity of good comparative data to support our hypothesis. We have highlighted several areas that are of importance to non-clinical researchers in the neuroscience community. We have also therefore amended our introduction and have removed our ‘proposal’.

We have made the statement referring to Kordower 2013 (ref 7, p3), more anatomically specific “By 4 years following diagnosis and thereafter, minimal, if any, tyrosine hydroxylase or dopamine transporter staining remained through- out the dorsal and mid-to-lateral aspects of the putamen (Figs 1D–L and 2D–L)”

We are unclear what the objection is regarding pathological studies in RBD.

We refer to the point that there is underdiagnosis of RBD given the diagnostic challenges and the fact that there may be an ascertainment bias in terms of gender in RBD. However, we are not aware of any data to reference this.

We are grateful for the pointer to the work done by Mahlknecht and colleagues. We have referenced the excellent 2015 Neurology paper in our discussion of anosmia in RBD (p6).
